# A multidimensional blood stimulation assay reveals immune alterations underlying systemic juvenile idiopathic arthritis

**DOI:** 10.1084/jem.20170412

**Published:** 2017-11-06

**Authors:** Alma-Martina Cepika, Romain Banchereau, Elodie Segura, Marina Ohouo, Brandi Cantarel, Kristina Goller, Victoria Cantrell, Emily Ruchaud, Elizabeth Gatewood, Phuong Nguyen, Jinghua Gu, Esperanza Anguiano, Sandra Zurawski, Jeanine M. Baisch, Marilynn Punaro, Nicole Baldwin, Gerlinde Obermoser, Karolina Palucka, Jacques Banchereau, Sebastian Amigorena, Virginia Pascual

**Affiliations:** 1 Baylor Institute for Immunology Research, Dallas, TX; 2 Institut National de la Santé et de la Recherche Medicale U932, Institut Curie, PSL Research University, Paris, France; 3 University of Texas Southwestern Medical Center, Dallas, TX; 4 The Jackson Laboratory for Genomic Medicine, Farmington, CT; 5 Texas Scottish Rite Hospital for Children, Dallas, TX

## Abstract

The etiology of sporadic human chronic inflammatory diseases remains mostly unknown. To fill this gap, we developed a strategy that simultaneously integrates blood leukocyte responses to innate stimuli at the transcriptional, cellular, and secreted protein levels. When applied to systemic juvenile idiopathic arthritis (sJIA), an autoinflammatory disease of unknown etiology, this approach identified gene sets associated with specific cytokine environments and activated leukocyte subsets. During disease remission and off treatment, sJIA patients displayed dysregulated responses to TLR4, TLR8, and TLR7 stimulation. Isolated sJIA monocytes underexpressed the IL-1 inhibitor aryl hydrocarbon receptor (AHR) at baseline and accumulated higher levels of intracellular IL-1β after stimulation. Supporting the demonstration that AHR down-regulation skews monocytes toward macrophage differentiation, sJIA monocytes differentiated in vitro toward macrophages, away from the dendritic cell phenotype. This might contribute to the increased incidence of macrophage activation syndrome in these patients. Integrated analysis of high-dimensional data can thus unravel immune alterations predisposing to complex inflammatory diseases.

## Abstract (precis)

The etiology of autoinflammation in systemic juvenile idiopathic arthritis is unclear. Cepika et al. use integrated analysis of multidimensional blood stimulation data, applied to patients while off treatment and in complete remission, to reveal underlying cellular and molecular mechanisms that might predispose to disease.

## Introduction

Human immune responses arise as the result of complex molecular and cellular interactions upon exposure to environmental and internal triggers. Comprehensive examination of these networks in health and disease has been facilitated by systems biology, a discipline that employs high-throughput assays to evaluate thousands of parameters simultaneously (Chuang et al., 2010; Kidd et al., 2014). Systems-level analysis of blood and/or affected tissues unraveled pathogenic mechanisms in complex diseases such as cancer, autoimmunity, and sterile inflammation (Chuang et al., 2010; Pascual et al., 2010; Werner et al., 2014). Immune cells are commonly profiled ex vivo, which represents a snapshot of their in vivo phenotype. Although well suited to characterize effector responses during active disease, ex vivo profiling often fails to reveal the underlying molecular phenotypes and events predisposing to disease development. For many multifactorial diseases, understanding their etiology and immunopathogenesis calls for complementary approaches that can identify triggers of inflammation and underlying immune alterations.

The functional capacity of immune cells can be probed in vitro by activating leukocytes with ligands that target specific inducible pathways. Whole-blood stimulation has the advantage of preserving complex interactions between leukocytes and plasma components present in vivo (Chaussabel et al., 2010), while avoiding the manipulation bias, cost, and time required to extract individual cell populations. In vitro blood stimulation was used to unveil defects in TLR and cytokine signaling in primary immunodeficiencies (Alsina et al., 2014), characterize responses to pathogenic stimuli and cytokines in healthy adults (Blankley et al., 2014; Duffy et al., 2014; Urrutia et al., 2016), or identify a lupus-specific chemokine signature (O’Gorman et al., 2015). However, these studies measured or analyzed transcription (Alsina et al., 2014; Blankley et al., 2014; Urrutia et al., 2016), secreted cytokines (Duffy et al., 2014), or cell-bound protein (O’Gorman et al., 2015) in isolation. Used independently, each method has limitations. The blood transcriptome is affected by differential cellular composition and does not accurately reflect protein abundance (Vogel and Marcotte, 2012); transcriptome and secretome profiling do not reveal the cellular origin of perturbations, whereas leukocyte phenotyping by flow or mass cytometry is constrained by the number of measurable parameters.

To overcome these limitations, we devised a blood stimulation assay that simultaneously captures inducible gene expression profiles, secreted proteins, and cell subset–specific activation markers. We integrated the resulting data layers with weighted gene coexpression network analysis (WGCNA), a method designed to extract and explore biological networks from high-dimensional data (Langfelder and Horvath, 2008). The assay was first validated using healthy adult blood challenged with a broad array of immune stimuli and subsequently applied to analyze inducible blood responses in children with systemic juvenile idiopathic arthritis (sJIA), a rare and severe IL-1–driven autoinflammatory disease of unknown etiology (Gurion et al., 2012). Integrative analysis of these multidimensional data exposed unique transcriptional modules linked to leukocyte subset–specific activation and skewing of cytokines milieus. In sJIA, leukocytes from patients in complete remission displayed dysregulated responses to TLR4, TLR8, and TLR7 stimulation, whereas monocytes were highlighted as potential drivers of inflammation. When differentiated in vitro, sJIA monocytes displayed a bias toward macrophage differentiation, which might contribute to the increased risk for macrophage activation syndrome (MAS) in this disease. Altogether, our experimental and analytical strategy could open the door to the development of preclinical screening assays and potentially early intervention in complex human inflammatory diseases.

## Results

### Blood transcriptional responses to innate stimuli in healthy adults

**Figure 1.**
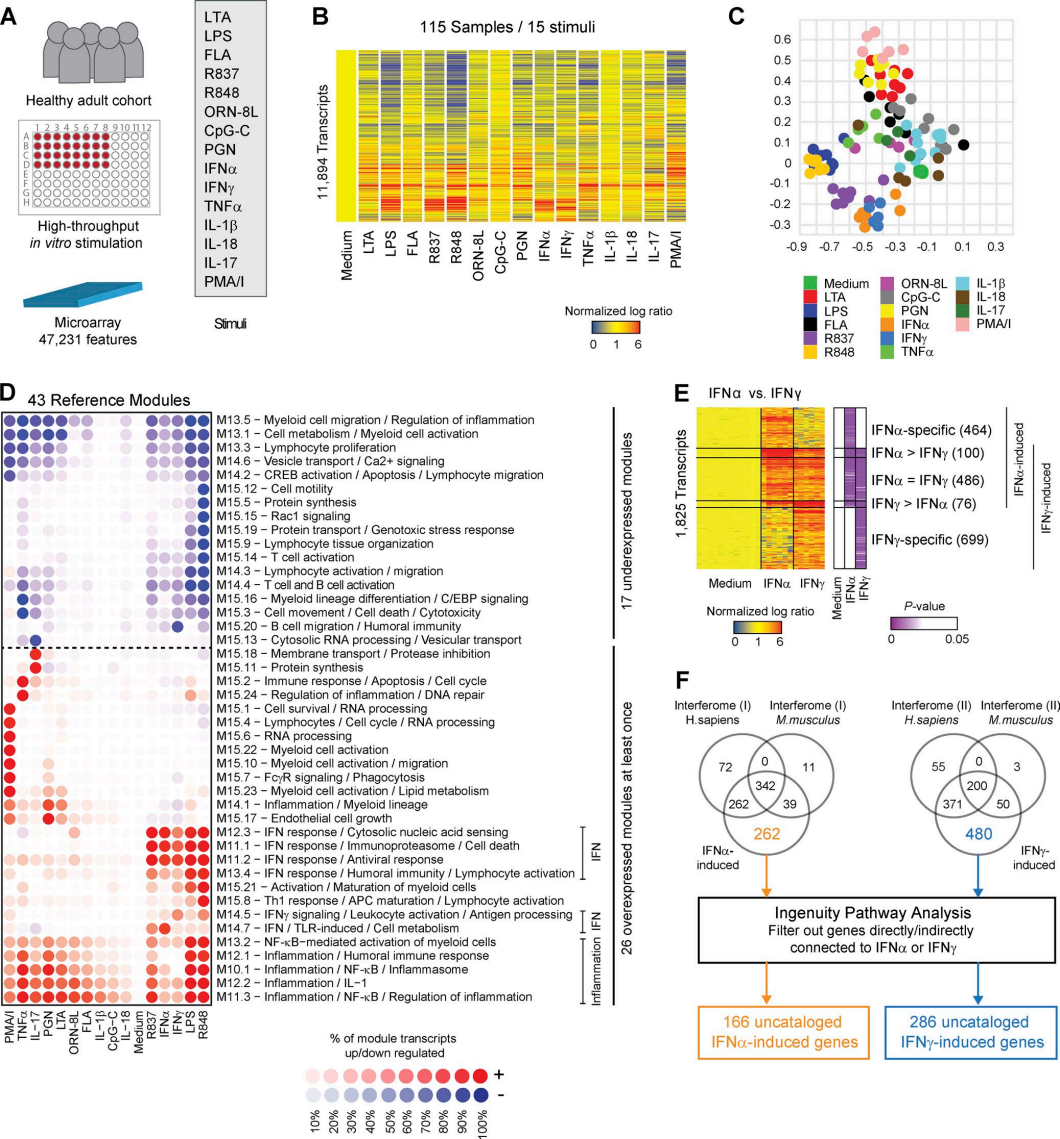
**Transcriptional landscape of healthy adult blood stimulated with 15 stimuli.** (A) Experimental workflow. (B) Hierarchical cluster of the 11,894 DETs in healthy adult blood cultured in vitro for 6 h in 16 conditions. Data are normalized to the medium control for each donor and averaged per stimulus. The median number of replicates per stimulus was eight, with an interquartile range of three. Samples from 13 donors were processed in five independent experiments. (C) Principal-component analysis based on the 11,894 DETs identified in B. Samples are colored by stimulus. (D) Hierarchical clustering of the reference module fingerprints. Module score is expressed as a percentage (transparency scale) of transcripts twofold over- (red) or underexpressed (blue). Data are normalized to the medium control for each donor. Fingerprints are averaged by stimulus. (E) Hierarchical clustering of the DETs in IFN-α versus IFN-γ stimulations. Induction was defined by significance analysis (ANOVA, P < 0.05) and normalized expression >1.25. When induced by both IFN-α and IFN-γ, transcripts were further separated based on the differential magnitude of expression using a twofold threshold (IFN-α = IFN-γ, IFN-α > IFN-γ, IFN-γ > IFN-α). (F) Identification of uncataloged type I and type II IFN-inducible transcripts. Venn diagrams represent the overlap between IFN-inducible transcripts in our dataset and in the INTERFEROME database, including both *H. sapiens* and *Mus musculus* organisms (normalized fold change >1.25). Transcripts uncataloged in the INTERFEROME were further subjected to IPA where transcripts connected directly or indirectly to IFNs were filtered out. IFN-α– and IFN-γ–induced transcripts were analyzed separately. PMA/I, PMA/ionomycin.

We first characterized the spectrum of transcriptional responses in healthy adult blood stimulated for 6 h with 15 ligands (Fig. 1 A). These included agonists for surface TLRs (TLR2, TLR4, and TLR5), intracellular TLRs (TLR7, TLR8, and TLR9) and NOD receptors, recombinant cytokines (IFN-α, IFN-γ, TNF-α, IL-1β, IL-17, and IL-18), and the protein kinase C activator PMA with the calcium ionophore ionomycin (Table S1). The 11,894 differentially expressed transcripts (DETs) were clustered (Fig. 1 B) and analyzed by principal-component analysis. The Gram-positive bacteria-derived ligands PGN and LTA clustered with PMA/ionomycin. IFN-α and IFN-γ clustered with IFN-inducing ligands, including LPS, R837, and R848 (Fig. 1 C). Clustering of R848 and LPS was not caused by LPS contamination (Fig. S1 A). Although oligonucleotides that stimulate TLR8 (ORN-8L) and TLR9 (CpG-C) induced an IFN response in PBMCs (Guiducci et al., 2013), this was not demonstrated in whole blood, where the response was predominantly proinflammatory (IL-1 and NF-κB pathways; Fig. S1 B). This may be caused by conformational or interaction alterations of oligonucleotides in plasma, because low-molecular-weight compounds that activate TLR7 (R837) and TLR8 (R848) induce IFN signatures in blood (Coch et al., 2013).

To facilitate the interpretation of these signatures, we constructed a modular framework of coexpressed transcripts (Chaussabel et al., 2008). The 43 modules obtained (hereafter “reference modules”) were annotated using a combination of knowledge-based and data-driven approaches, including Ingenuity Pathway Analysis (IPA), gene ontology, INTERFEROME (Rusinova et al., 2013; Fig. S1, C and D), and PASTAA (Roider et al., 2009) upstream transcription factor binding site enrichment analyses (Table S2). Modular fingerprints were derived for each stimulus and hierarchically clustered (Fig. 1 D). Five modules were associated with NF-κB–mediated inflammation and the inflammasome and six with IFN signaling. Several modules were specific for IFN-γ, TNF-α, or IL-17. These modules highlight the spectrum of blood transcriptional responses to innate stimuli and can act as reference gene sets for downstream analyses.

This dataset can also be used to identify novel components in specific immune pathways. For example, transcripts differentially induced by IFN-α and IFN-γ (Fig. 1 F; http://tollgene.org) overlapped with the INTERFEROME database, a manually curated repository of IFN-regulated genes (Rusinova et al., 2013). Transcripts not associated with either type I or II IFN in the INTERFEROME were further analyzed with IPA. This yielded 166 IFN-α– and 286 IFN-γ–induced transcripts not previously cataloged in these reference databases (Table S3). Overall, the signatures obtained after in vitro perturbation with a broad array of ligands delineate common and specific components of the human blood transcriptional responses to stimuli and represent an extensive resource to characterize inducible immune networks.

### Assay expansion to cellular and secreted protein responses

**Figure 2.**
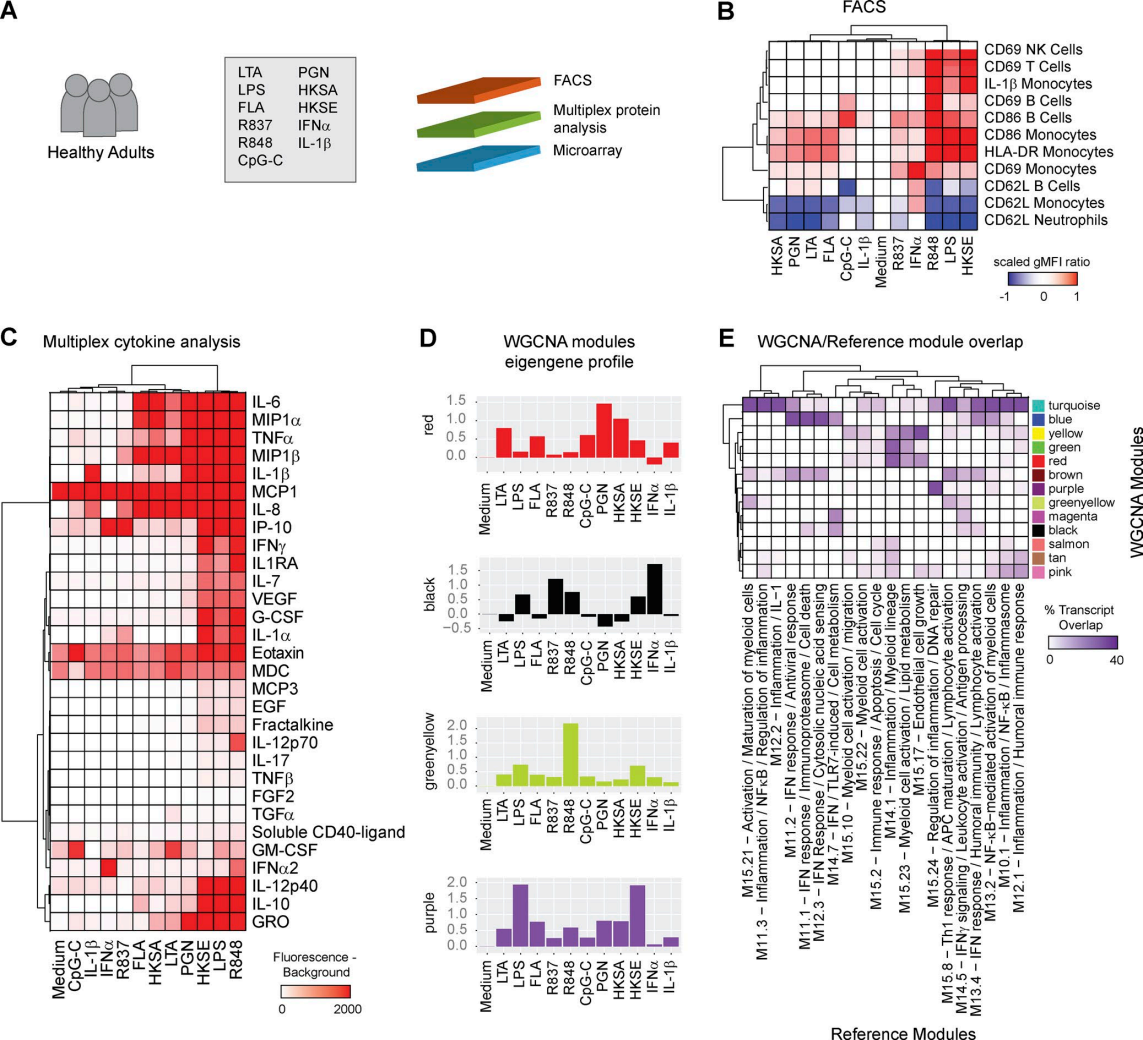
**Multidimensional assessment of healthy adult blood responses to stimulation by microarray, FACS, and multiplex cytokine analysis.** (A) Experimental workflow. (B) Hierarchical cluster of surface and intracellular proteins measured by FACS after 6-h stimulation. gMFI ratios were scaled per marker and cell subset by setting the medium reference control to 0 and the condition with maximum change to 1 or −1 (up or down). (C) Hierarchical clustering of 30 secreted proteins measured by Luminex. (D) Eigengene profiles of four WGCNA modules. The data represent log2-transformed ratios of stimulated samples normalized to medium control. (E) Heatmap representing the transcript overlap between WGCNA modules and the reference modules from Fig. 1 D. HKSA, heat-killed *Staphylococcus aureus*; HKSE, heat-killed *Salmonella enterica*.

Although transcriptional profiling of fresh blood prevents confounding factors introduced by cell subset isolation and/or cryopreservation, the signatures of dominant cell populations may mask those of less abundant subsets. We therefore expanded the assay in three healthy adults to examine cell subset–specific activation and inflammasome triggering by FACS (Fig. 2 A and Fig. S2 A). After 6-h stimulation, monocytes, neutrophils, NK cells, B cells, and T cells were stained for surface expression of CD62L, CD69, CD86, and HLA-DR and intracellular expression of IL-1β. To maximize the range of stimuli per sample, we limited the blood aliquot for FACS to 50 µl and excluded low-frequency populations from the analysis. The gating strategy is summarized in Fig. S2 (B and C).

Of the examined subsets, TLR9 stimulation only activated B cells. Neutrophil activation, measured by CD62L shedding, paralleled CD86 and HLA-DR up-regulation on monocytes. CD69 expression on T cells and NK cells increased predominantly in conditions that also induced inflammasome activation. IFN-α had unique effects on monocytes, strongly up-regulating surface CD69 and CD62L, whereas all other stimuli induced CD62L shedding. Both CD69 and CD62L are involved in leukocyte trafficking. CD69 inhibits the sphingosine 1 phosphate receptor, preventing leukocyte egress from lymph nodes during immune responses (Shiow et al., 2006; Cyster and Schwab, 2012). CD62L regulates leukocyte migration to lymph nodes and inflammation sites (Ivetic, 2013). These results suggest a differential regulation of monocyte and lymphocyte trafficking by IFN and TLR ligands (Fig. 2 B and Fig. S3).

In parallel, we measured the concentration of 41 secreted proteins by Luminex, 30 of which were detectable in at least one condition (Fig. 2 C; http://tollgene.org). Protein secretion patterns overlapped between many TLR ligands, but certain stimuli elicited distinct profiles, which illustrated the functional outcomes linked to inducible gene networks. For example, R848, despite clustering tightly with LPS at the transcript level (Fig. 1, C and D), induced high levels of IFN-α and IL-12p70 secretion that were not elicited by LPS. IFN-α induced the expression of 1,339 transcripts (http://tollgene.org), but IP-10 was the only abundantly secreted protein out of the 41 measured. In addition, levels of secreted IL-1β highly correlated with intracellular IL-1β expression as well as with *IL1B* transcript levels. Intracellular IL-1β detection by FACS was further validated by Western blot in isolated monocytes (Fig. S4).

To identify transcriptional pathways associated with cellular activation and secreted cytokine profiles, we conducted WGCNA (Langfelder and Horvath, 2008). Briefly, this method (1) extracts coexpression modules, (2) correlates the mean expression profile of each module (eigengene) with continuous variables such as surface marker geometric mean fluorescence intensity (gMFI) ratios or secreted protein concentrations, and (3) identifies major transcriptional hubs associated with continuous variables through gene significance (GS) and module membership (MM) analysis.

**Figure 3.**
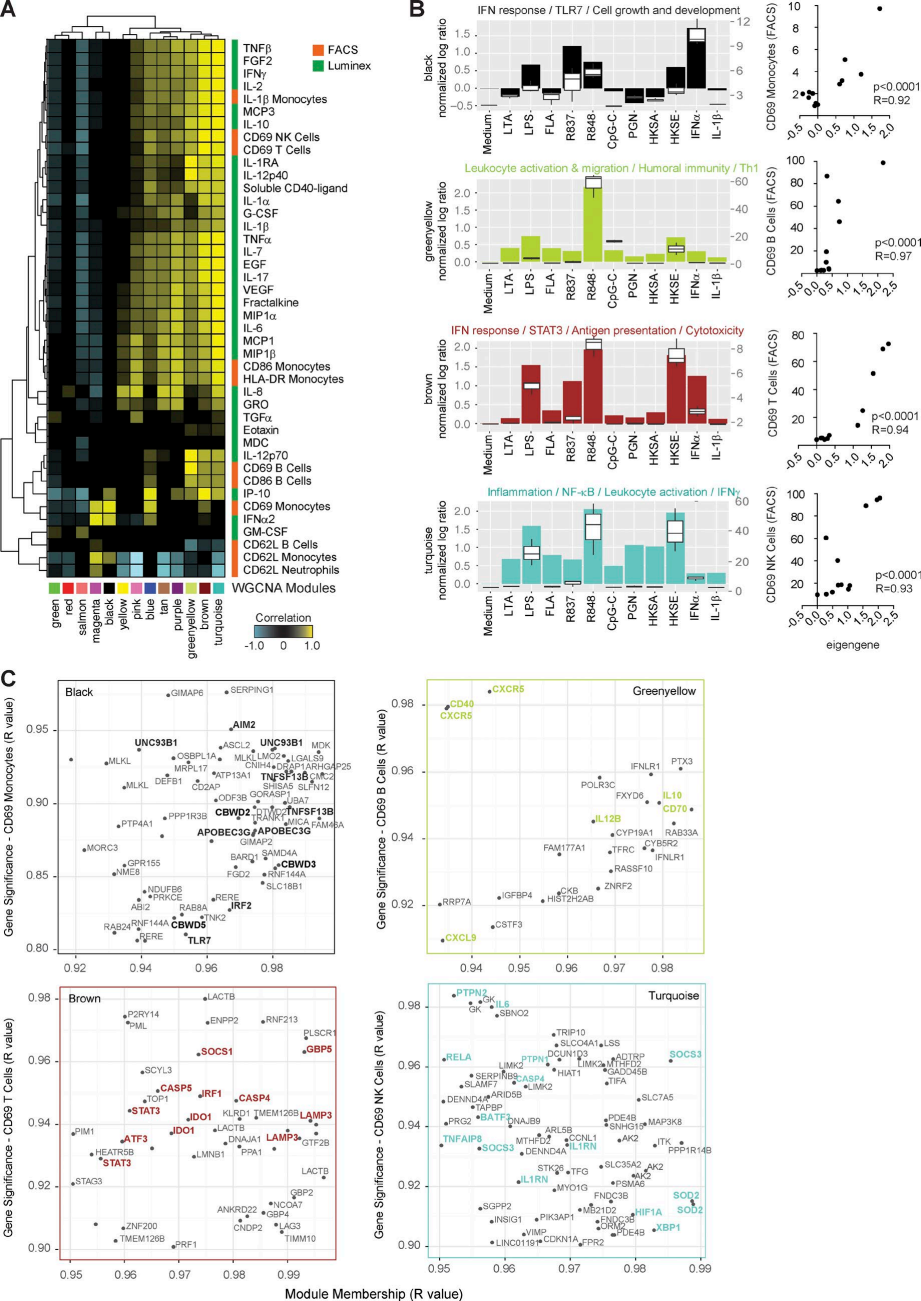
**Integration of microarray, FACS and multiplex cytokine measurements by weighted gene coexpression network analysis.** (A) Hierarchical cluster of the correlation matrix between WGCNA module eigengenes (x axis) and FACS (orange) or Luminex (green) measurements (y axis). (B) Area charts representing the module eigengene for four modules linked to leukocyte subset activation as quantified by CD69 expression. Charts were overlaid with boxplots representing the eigengene’s best FACS or Luminex correlate. Pearson correlations between eigengenes and protein measurements are represented as x–y charts on the right. (C) x–y plots representing the MM (x axis, R value) versus GS (y axis, R value) analysis for the four modules from B and their best protein correlate. MM and GS represent the correlation of each gene to the module eigengene and the protein expression, respectively. Genes highly representative of the module functional annotation were highlighted in bold font. Genes are duplicated when several probes were detected within the selected range of MM and GS. HKSA, heat-killed *Staphylococcus aureus*; HKSE, heat-killed *Salmonella enterica*.

WGCNA identified 13 uniquely color-coded modules with distinct expression profiles across stimuli (Fig. 2 D; http://tollgene.org). To functionally interpret these, we quantified their transcript overlap with the annotated reference modules (Fig. 2 E). Thus, the reference modules serve as a template to rapidly infer the biological function of WGCNA modules. A correlation matrix between module eigengenes, FACS, and Luminex measurements was then built to associate transcript- and protein-level changes (Fig. 3 A). This enabled, for example, the identification of transcriptional networks linked to CD69 induction in distinct leukocyte subsets. On monocytes, CD69 correlated with the module predominantly induced by IFN-α (black), and clustered with soluble IFN-α2. On B cells, CD69 correlated with the leukocyte activation and migration module (greenyellow, strongly induced by R848), and clustered with soluble IL-12p70. On T cells and NK cells, CD69 correlated with the IFN response (brown) and inflammation–NF-κB (turquoise) modules, both induced by LPS, R848, and heat-killed *Salmonella enterica* and clustered with soluble IFN-γ, IL-2, and IL-10 (Fig. 3 B).

To identify specific transcripts associated with leukocyte activation markers, we conducted GS and MM analysis. GS quantifies the correlation between a gene and a protein trait of interest (the higher the absolute GS value, the higher the biological significance of the gene), whereas MM quantifies the fit of a gene within its module and indicates its connectivity level with other genes within the module. We focused on transcripts that highly correlated (R > 0.80) both with protein expression and module eigengene (Fig. 3 C). Monocyte CD69 expression closely correlated with transcripts involved in intracellular nucleic acid response such as *TLR7*, *AIM2*, *APOBEC3G*, *UNC93B1*, and *IRF2*, all of which are IFN inducible. The B cell activation genes *CD40*, *CD70*, *CXCR5*, *IL10*, and *IL12B* paralleled CD69 up-regulation on B cells. T cell and NK cell CD69 expression correlated with the IFN-inducible transcripts *IRF1*, *STAT3*, *LAMP3*, *SOCS1*, and *IDO1* (brown module) and the proinflammatory transcripts *IL6*, *RELA*, *CASP4*, and *HIF1A* (turquoise module), respectively. GS and MM analysis confirmed the previous results, identifying distinct hub genes that correlated with CD69 expression on different leukocyte subsets.

Overall, analytical integration of multidimensional stimulation data can delineate transcriptional and cytokine profiles associated with the activation of individual cell subsets in blood, thus providing a platform to expand the characterization of inducible immune networks.

### Increased inflammatory responses to TLR4 and TLR8 ligands in sJIA patients

**Figure 4.**
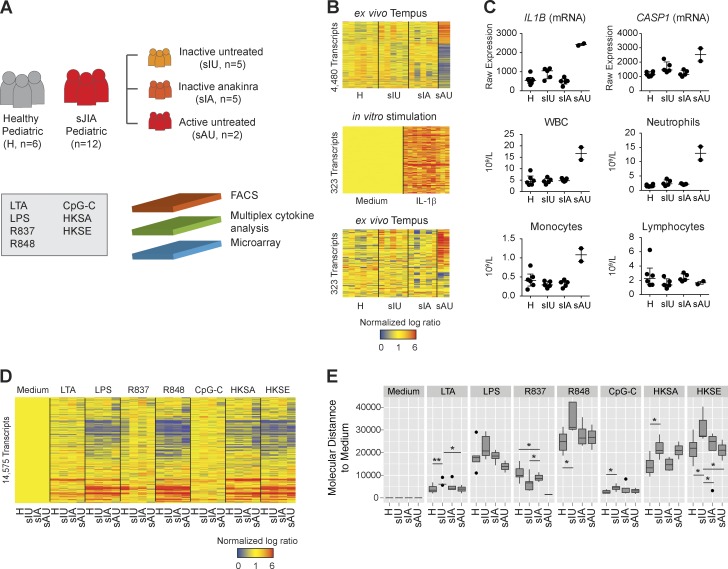
**Hyperresponsiveness to TLR4 and TLR8 ligands in inactive untreated sJIA patients.** (A) Experimental workflow. (B) Hierarchical cluster of the 4,480 DETs between healthy controls and sJIA patient groups ex vivo (baseline; top). Hierarchical cluster of the 323 transcripts overexpressed ≥1.5-fold in healthy blood stimulated with IL-1β for 6 h (ref. Fig. 1; center). Data are normalized to medium controls for each donor. Hierarchical cluster of the same 323 transcripts in ex vivo signatures (bottom). Ex vivo blood samples were processed in two independent experiments (C) Dot plots representing the raw expression of *IL1B* and *CASP1* transcripts, white blood cells (WBC), neutrophil, monocyte, and lymphocyte absolute counts, displayed by patient group. Horizontal lines indicate the median, whiskers the interquartile range. (D) Hierarchical cluster of the 14,575 DETs in stimulated blood from sJIA patients and pediatric healthy controls. Samples from 18 donors were processed in 16 independent experiments. (E) Box plot representing the molecular distance to medium (MDTM) derived from the 14,575 DETs identified in D, displayed per stimulus and patient group. The MDTM is calculated for each sample as the sum of absolute normalized fold changes ≥2 in the list of transcripts considered. Horizontal lines indicate the median. Boxes represent the interquartile range, and whiskers nonoutlier range (H, healthy). HKSA, heat-killed *Staphylococcus aureus*; HKSE, heat-killed *Salmonella enterica*.

We then applied our assay to identify altered inducible immune networks in sJIA. Blood was obtained from five inactive untreated patients (sIU), five inactive patients treated with recombinant IL-1R antagonist anakinra (sIA), two active untreated (sAU) patients, and six demographically matched healthy children (Fig. 4 A). Because the frequency and activation state of leukocytes influence stimulation outcomes, we first analyzed the ex vivo gene expression and complete blood counts (CBCs). Although signatures and cell composition from sIU and sIA patients were comparable to those of healthy controls, sAU patients displayed a proinflammatory signature enriched for IL-1β–inducible transcripts (*IL1B* and *CASP1*) and demonstrated leukocytosis, neutrophilia, and monocytosis (Fig. 4, B and C). We subsequently focused our analysis on sIU patients to reveal inducible molecular alterations not confounded by activation state or treatment.

Although 14,575 DETs were identified after 6-h challenge with seven stimuli (Fig. 4 D), no significant DETs were detected by ANOVA between inactive patients and healthy controls after *post hoc* test and false discovery rate correction. However, the molecular distance to medium (MDTM), a composite score reflecting the global intensity of the signature in each sample, revealed increased transcriptional activity in sIU patients in response to LPS, R848, and heat-killed bacteria (*Staphylococcus aureus* and *Salmonella enterica*; Table S1), suggesting transcriptional hyperresponsiveness to TLR4 and TLR8 ligands (Fig. 4 E).

**Figure 5.**
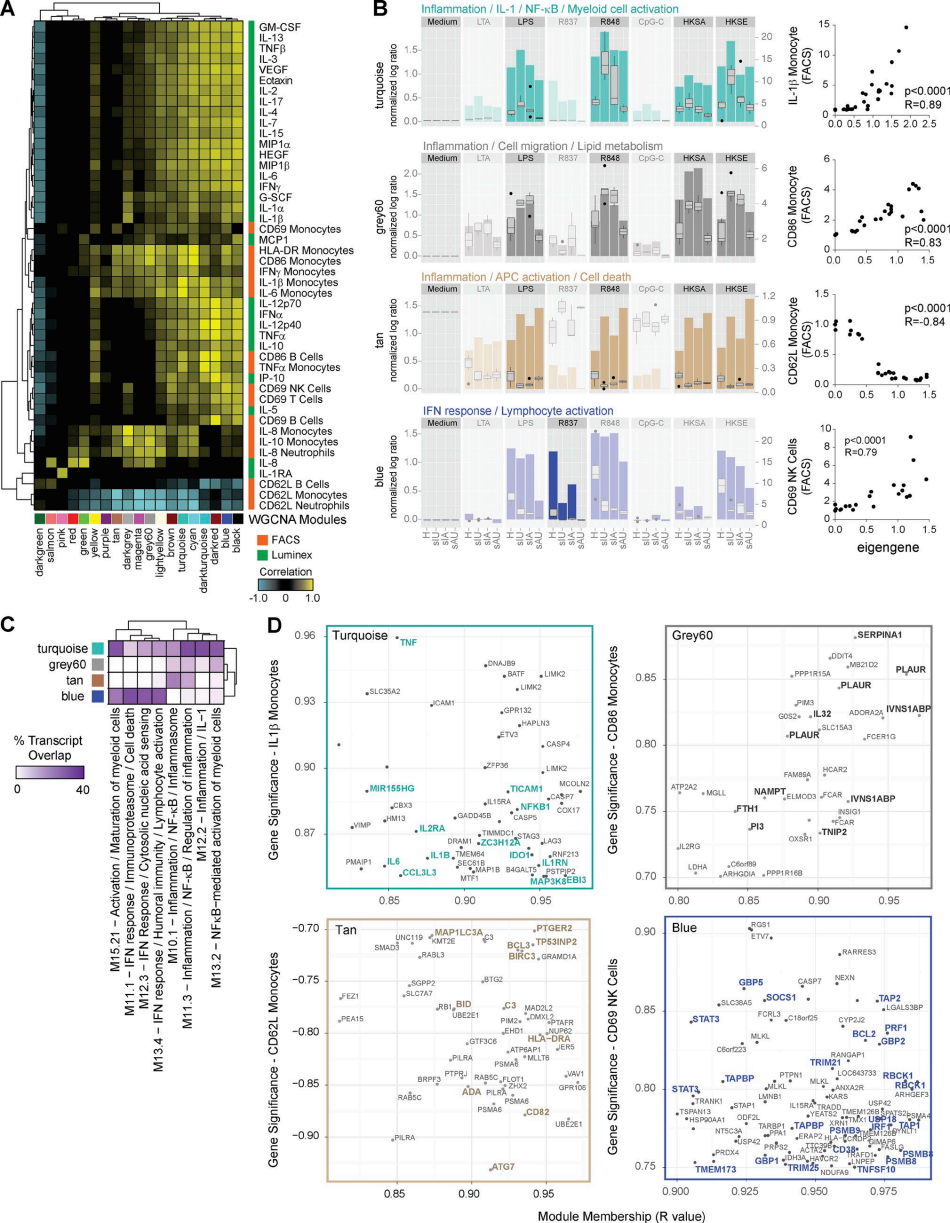
**Loss of balance between proinflammatory and IFN responses in sJIA patients.** (A) Hierarchical cluster of the module–trait correlation matrix obtained by WGCNA in stimulated sJIA and control blood dataset. (B) Bar charts representing the eigengene profiles of four WGCNA modules that display distinct transcriptional differences between healthy controls and sJIA patient groups. Bar charts are overlaid with box plots of the eigengenes’ best FACS correlate (right; y axis). Pearson correlations between eigengenes and protein measurements are represented as x–y charts on the right (H, healthy). (C) Hierarchical cluster of the overlap between WGCNA modules and the reference modules. (D) x–y plots representing the MM (x axis) versus GS (y axis) analysis for the four modules from B and their best protein correlate. HKSA, heat-killed *Staphylococcus aureus*; HKSE, heat-killed *Salmonella enterica*.

Of the 19 modules identified by WGCNA (Fig. 5 A), we focused on the ones that strongly correlated with protein markers and that displayed transcriptional differences between sIU patients and healthy controls in response to any stimulus (Fig. 5 B). Modules were annotated by overlapping their transcriptional content with that of the reference modules (Fig. 5 C). Three modules induced by LPS, R848, and heat-killed bacteria correlated with monocyte activation markers and were overexpressed in sJIA patients compared with healthy donors. The inflammation–IL-1–NF-κB module (turquoise) was overexpressed in sIU patients only and best correlated with the expression of intracellular IL-1β (P = 0.89) and IL-6 (P = 0.89) in monocytes. The inflammation–cell migration module (grey60) was overexpressed in sIU and sIA patients and correlated best with surface CD86 (R = 0.83) and HLA-DR (0.80) on monocytes. The inflammation/APC activation module (tan) was overexpressed in sIU and sAU patients and correlated inversely with monocyte CD62L expression (R = −0.84). Conversely, the IFN response–lymphocyte activation module (blue) correlated with CD69 expression in T cells and NK cells (R = 0.79) and was underexpressed in inactive sJIA patients in response to TLR7 stimulation. As observed in vivo (Quartier et al., 2011), anakinra treatment reduced IL-1–mediated inflammation in vitro (turquoise module) and partially restored TLR7-induced IFN response (blue) but had no effect on monocyte maturation and migration (grey60).

To identify transcripts highly correlated with the activation of leukocyte subsets in sJIA, we again conducted GS versus MM analysis (Fig. 5 D). Transcripts encoding major proinflammatory cytokines (*IL1B*, *IL6*, *TNF*, and *EBI3*), TLR signaling components (*NFKB1*, *TICAM1*, and *MAP3K8*) and inflammation regulators (*MIR155HG* and *IL1RN*) closely aligned with monocyte IL-1β production. Monocyte CD86 and HLA-DR expression correlated with transcripts encoding IL-6–inducing cytokines (*IL32* and *NAMPT*), antimicrobial proteins (*PI3* and *FTH1*), and immune regulators (*TNIP2* and *IVNS1ABP*). CD62L shedding from monocytes was accompanied by up-regulation of genes involved in co-stimulation (*CD82*, *ADA*, and *HLADRA*), autophagy (*ATG7*, *TP53INP2*, and *MAP1LC3A*) and NF-κB signaling (*BIRC* and *BCL3*). Finally, NK and T cell activation was paralleled by induction of IFN response (*TMEM173*, *IRF1*, *USP18*, *TRIM25*, *STAT3*, and *GBP2*), perforin (*PRF1*), immunoproteasome (*PSMB8* and *PSMB9*), and antigen-presentation pathways (*TAP1*, *TAP2*, and *TAPBP*).

Altogether, our assay detected transcriptional and cellular hyperresponsiveness to inflammasome-inducing TLR4 and TLR8 ligands and hyporesponsiveness to IFN-inducing TLR7 ligands in sJIA patients in remission. These results support an underlying disequilibrium between proinflammatory and IFN pathways in sJIA that may contribute to disease onset and flares.

### A web interface to enable data dissemination and exploration

To facilitate data access and support further discovery, we developed an interactive web interface (available at http://tollgene.org). Users can (1) see an overview of the assay, with relevant reagents and a detailed protocol; (2) filter DETs for each stimulus in healthy controls using customizable parameters; (3) compare transcriptional signatures using Venn diagrams; (4) analyze module fingerprints and transcript content; (5) visualize FACS and Luminex data in healthy controls and sJIA patients; and (6) analyze WGCNA output in healthy controls and sJIA patients.

### Reduced expression of inflammation regulators and increased IL-1β production in isolated sJIA monocytes

**Figure 6.**
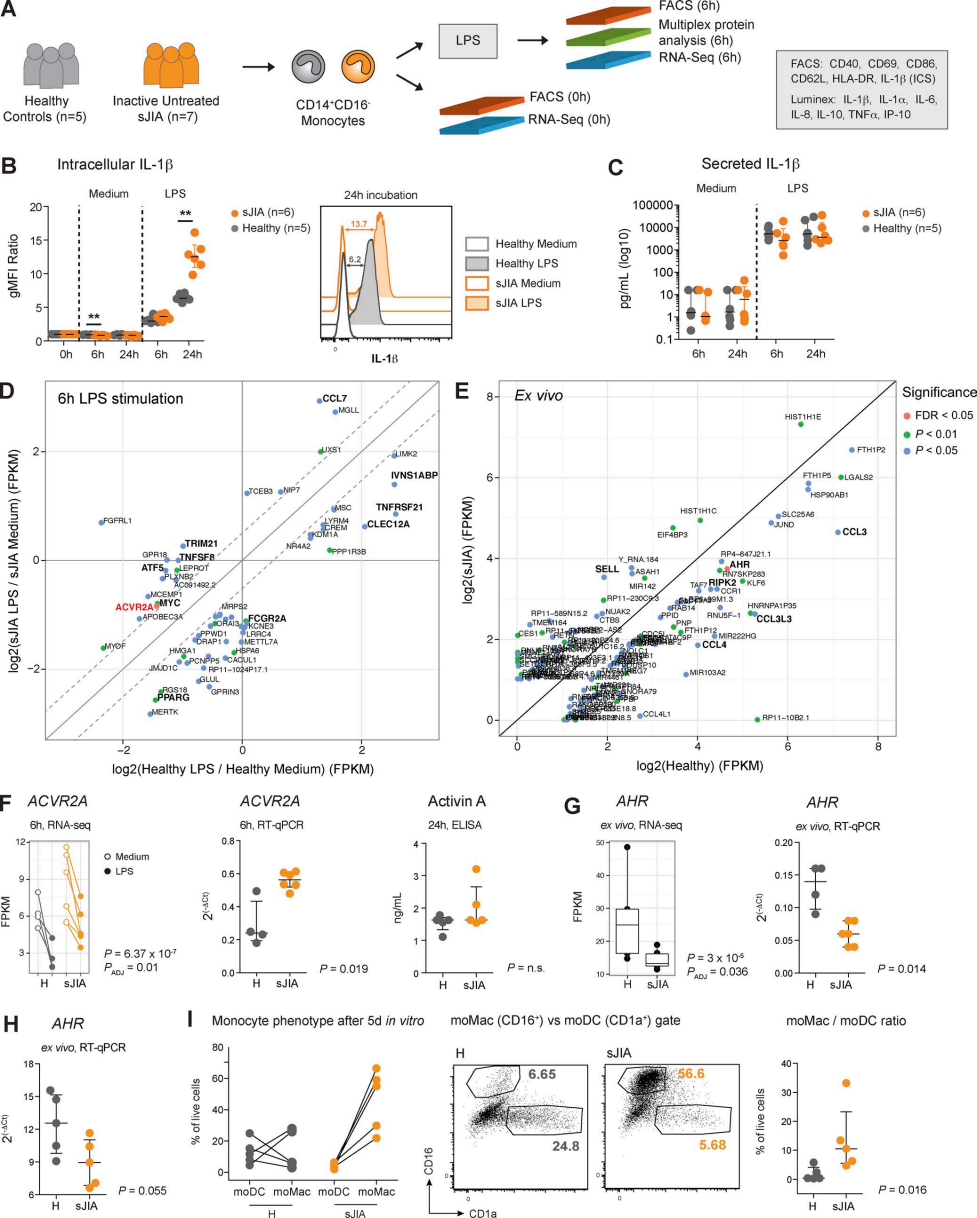
**Monocytes from sIU patients accumulate IL-1β after LPS stimulation, underexpress AHR gene at baseline, and differentiate into macrophages in vitro.** (A) Experimental workflow. Monocytes from each donor were isolated and cultured independently. (B) gMFI ratios of intracellular IL-1β in monocytes for indicated conditions (left). Data are normalized to each donor’s baseline gMFI. **, P < 0.01, Mann–Whitney test. Histogram overlay of intracellular IL-1β in representative healthy and sJIA monocytes (right). (C) Concentration of secreted IL-1β in the supernatants of cultured monocytes. (D) x–y chart of DEGs between stimulated sJIA and healthy monocytes. Values represent FPKM ratios of healthy LPS to healthy medium (x axis) versus sJIA LPS to sJIA medium (y axis). Genes with P < 0.05 were filtered for an absolute log2(ratio) difference ≥1.5. (E) x–y chart of DEGs between sJIA and healthy monocytes ex vivo. Genes with P < 0.05 were filtered for an absolute log2 difference sJIA-healthy ≥1.5. Genes with absolute log2(ratio) (D) or log2 (E) FPKM values <1 were removed. (F) Expression of *ACVR2A* gene measured by RNA-seq and quantitative RT-PCR. The levels of activin A protein in supernatants of monocytes stimulated for 24 h with LPS are shown on the right. Activin A was not detectable in unstimulated conditions or at 6 h LPS. (G) Expression of *AHR* gene measured by RNA-seq and quantitative RT-PCR. (H) Expression of *AHR* gene measured by quantitative RT-PCR in an independent cohort of sJIA patients and controls (*n* = 5). (I) Monocytes from the second cohort were differentiated in vitro for 5 d; the phenotype of monocyte-derived cells was determined by flow cytometry as macrophage (moMac; CD16^+^CD1a^−^) or DC (moDC; CD16^−^CD1a^+^; left). Representative plots from one healthy control and one patient are shown in the middle. The ratio of the percentage of moMac cells to the percentage of moDC cells is shown on the right. Cultures were performed in three independent experiments. H, healthy. In dot plots, horizontal lines indicate the median and whiskers the interquartile range. In box plots, horizontal lines indicate the median, boxes the interquartile range, and whiskers the nonoutlier range. P-values for flow cytometry and quantitative RT-PCR data were calculated using Mann–Whitney *U* test.

Most of the dysregulated pathways that were identified in our assay involved monocytes. In addition, these cells are the only blood mononuclear cells expressing the three TLRs that triggered differential transcriptional responses in patients. To further understand monocyte alterations in sJIA, we sorted CD14^+^CD16^−^ monocytes from cryopreserved PBMCs from seven sIU patients and five healthy pediatric controls. We profiled their transcriptome by RNA sequencing (RNA-seq) ex vivo and after 6-h culture with LPS. Monocyte activation and cytokine production were measured at 6 and 24 h by FACS and Luminex, respectively (Fig. 6 A and Fig. S5 A). As in cultured blood, sJIA monocytes displayed increased HLA-DR levels after 6-h culture with LPS (Fig. S5 B). Intracellular IL-1β expression in response to LPS was comparable in isolated patient and healthy monocytes at 6 h. At 24 h, however, sJIA monocytes accumulated significantly more intracellular IL-1β (Fig. 6 B). Increased intracellular production of IL-1β did not result in increased secretion of the mature protein compared with controls at both time points (Fig. 6 C). To confirm this result, we repeated the experiment using monocytes from an independent cohort of patients and controls (*n* = 4 for each group), including additional 48-h and 72-h time points (not depicted) and observed similar levels of secreted IL-1β in patients and controls.

Transcriptionally, 57 genes were differentially expressed between LPS-stimulated sIU and healthy monocytes at 6 h, and 119 genes were differentially expressed between sIU and healthy monocytes ex vivo (Table S4 and Fig. 6, D and E; unadjusted P < 0.05). At 6 h, LPS-stimulated sJIA monocytes displayed impaired down-regulation of *ACVR2A*, which encodes activin receptor type 2A. Increased expression of *ACVR2A* in conjunction with normal levels of its ligand (Fig. 6 F) may result in a net increase of activin receptor signaling, which was shown to inhibit IL-1β secretion without affecting its intracellular levels (Ohguchi et al., 1998).

LPS-stimulated sJIA monocytes underexpressed the *IVNS1ABP* gene (Fig. S5 C), which encodes the aryl hydrocarbon receptor (AHR) signaling enhancer (Dunham et al., 2006). In line with this, sJIA monocytes ex vivo expressed decreased levels of the *AHR* gene (Fig. 6 G) and its chaperone (Fig. S5 D). Besides its well-known function in xenobiotic metabolism (Stockinger et al., 2014), AHR was recently shown to be an important regulator of IL-1β–mediated inflammation (Bessede et al., 2014; Huai et al., 2014). In addition, AHR-deficient mouse macrophages are hyperresponsive to LPS (Kimura et al., 2009), and loss of AHR alters macrophage polarization (Climaco-Arvizu et al., 2016).

As sJIA patients are predisposed to macrophage activation syndrome (Ravelli et al., 2012), we next explored the phenotype of isolated sJIA monocytes after in vitro differentiation using a system that allows for monocytes to develop into macrophages and DCs in the same culture (Goudot et al., 2017). We first measured the expression of the *AHR* gene in an independent cohort of sJIA patients and healthy controls (*n* = 5), and we confirmed lower baseline *AHR* expression in sJIA monocytes (Fig. 6 H). After 5 d in culture, healthy monocytes differentiated comparably into DCs and macrophages, whereas sJIA monocytes acquired predominantly a macrophage phenotype (Fig. 6 I and Fig. S5 F).

Overall, sJIA monocytes isolated from asymptomatic untreated patients express lower levels of inflammatory regulators at baseline and develop an exacerbated proinflammatory phenotype after activation. Decreased AHR expression in sJIA monocytes might skew the differentiation of these cells toward macrophages and contribute in vivo to MAS, a potentially fatal complication.

## Discussion

We developed a multidimensional blood stimulation assay to identify altered inducible immune networks and to provide new insights into pathogenic mechanisms underlying complex inflammatory diseases. Our analytical strategy is the first to integrate data from gene expression, FACS, and multiplex cytokine readouts concurrently after challenge of blood cells with a broad array of stimuli. This integrated approach has several advantages. Comparing sets of coexpressed genes (modules) can identify subtle but relevant transcriptional differences undetectable by traditional gene-level statistical approaches (Haining and Wherry, 2010). Linking module expression to secreted proteins can uncover functional outcomes of transcriptional network activation. Finally, associating modules to blood leukocyte subset profiles can reveal specific cell populations involved in response to stimulation. In healthy adults, we show how dissecting inducible pathways leads to discovery of novel immune response components. These data represent a valuable resource that can be further explored through a user-friendly interface at http://tollgene.org. In sJIA, we detected underlying alterations in TLR signaling and skewing of the monocyte phenotype in patients otherwise clinically and transcriptionally comparable to healthy.

sJIA presents in childhood with fever, rash, elevated levels of acute phase proteins, leukocytosis, and serositis, which are accompanied or followed by debilitating arthritis (Gurion et al., 2012). The systemic presentation of sJIA and the excellent response to pharmaceutical IL-1 inhibition (Pascual et al., 2005; Quartier et al., 2011; Ruperto et al., 2012; Ilowite et al., 2014) are shared with several monogenic autoinflammatory diseases (Jesus and Goldbach-Mansky, 2014). However, these diseases are caused by genetic mutations within the inflammasome–IL-1 pathways, none of which have been found so far in sJIA. Furthermore, ex vivo profiling of circulating leukocytes in sJIA did not point toward a specific driver of inflammation, and a similar signature is present in other inflammatory diseases (Allantaz et al., 2007).

Stimulation of sJIA blood revealed a shift toward increased proinflammatory responses elicited by IL-1–inducing TLR4 and TLR8 ligands and a concurrent decrease in TLR7 and IFN responses. The antagonism between IFN-α and IL-1β signaling is well described (Guarda et al., 2011) and further substantiated in vivo by the induction of an IFN signature in the blood of sJIA patients after anakinra treatment (Quartier et al., 2011). Notably, differences between sJIA and healthy control responses were best revealed using blood from patients in remission and off treatment, as blood from active untreated patients displayed elevated baseline inflammatory signatures and was transcriptionally hyporesponsive to stimulation. Even though inactive untreated patients from our cohort did not show any signs of active disease detected by clinical examination, laboratory tests or ex vivo whole-transcriptome analysis, we cannot exclude the possibility that the observed dysregulated responses are caused by underlying low-grade inflammation as opposed to cell-intrinsic alterations.

Serum from active sJIA patients induces inflammation in healthy cells after in vitro incubation. This proinflammatory capacity correlates with disease activity (Pascual et al., 2005). Accordingly, the effect of serum from inactive untreated patients on healthy monocytes is comparable to that of healthy control serum (unpublished data). However, the blood environment seems to enhance specific monocyte responses. Intracellular levels of IL-1β were increased in sJIA monocytes after 6-h stimulation of whole blood. At the same time point, isolated sJIA monocytes cultured in FBS-enriched medium produced levels of IL-1β comparable with those of healthy cells. An increase in IL-1β production in isolated sJIA monocytes was not detected until 24 h. Delayed accumulation of intracellular IL-1β in isolated cells complemented independent observations in 24-h PBMC culture (Macaubas et al., 2012). Interestingly, secretion of IL-1β was comparable between asymptomatic patients and healthy controls in both blood and isolated monocytes. Proper regulation of IL-1β secretion in these inactive untreated patients may explain their quiescent clinical phenotype, despite exacerbated monocyte responses after stimulation.

Comparison of sJIA and healthy monocyte transcriptomes ex vivo and after stimulation with LPS did not provide a definite explanation for the divergence of IL-1β production and secretion in patients, partly because the exact mechanism of IL-1β secretion is still under debate (Piccioli and Rubartelli, 2013). However, transcriptome analysis identified genes that play a major role in regulating immune responses. LPS-stimulated sJIA monocytes displayed altered down-regulation of *ACVR2A*, which encodes a receptor for the immunomodulatory TGF-β family member activin A (Phillips et al., 2009). In stimulated human monocytes, activin A did not affect intracellular pro–IL-1β but inhibited the secretion of the mature protein, possibly by blocking caspase-1–mediated cleavage of the pro form (Ohguchi et al., 1998). Enhanced activin A receptor signaling in sJIA monocytes could explain the observed discrepancy between increased accumulation of intracellular IL-1β and secreted protein levels comparable to those of healthy monocytes.

Ex vivo, sJIA monocytes underexpressed *AHR* and its chaperone, *HSP90AB1*. AHR, a transcription factor known for promoting immune tolerance (Stockinger et al., 2014), inhibits *NLRP3* transcription and subsequent IL-1β secretion (Huai et al., 2014) and protects against endotoxin shock (Bessede et al., 2014), and AHR-deficient macrophages secrete more proinflammatory cytokines (Kimura et al., 2009). When differentiated in vitro, AHR-deficient sJIA monocytes predominately developed into macrophages, away from a DC phenotype. This skewing may contribute to the predisposition of sJIA patients to develop MAS, which is characterized by accumulation of activated hemophagocytic macrophages and overproduction of proinflammatory cytokines and often triggered by infections (Grom et al., 2016). The cause of altered AHR expression in sJIA remains unknown. Down-regulation of its enhancer, *IVNS1ABP*, in response to in vitro activation points toward epigenetic regulation, which could result from environmental stimuli such as pollutants or dietary compounds or microbiome-derived endogenous ligands (Cella and Colonna, 2015). As MAS is the main cause of mortality in sJIA, occurring overtly in 10% and subclinically in 30–40% of sJIA patients (Minoia et al., 2014), exploring the link to AHR may lead to novel diagnostic and therapeutic options.

Our approach can be tailored to study other immune diseases and conditions by adjusting the ligand panel, phenotypic markers, secreted analytes, technological platforms, and incubation times. Of note, incubation periods in blood should be carefully selected, as longer cultures are confounded by neutrophil apoptosis (Majewska et al., 2000), platelet activation, and secondary signals, whereas early time points display limited changes in protein expression. The blood volume can be scaled down to make it amenable for pediatric studies, where large blood samples are difficult to obtain. The assay could be further exploited to characterize the heterogeneity of innate immune responses in healthy individuals, predict responses to vaccines and/or adjuvants, and uncover novel molecular pathways that could facilitate disease monitoring and diagnosis. Overall, this combination of experimental, methodological, and computational resources could enable the dissection of underlying pathogenic mechanisms in a large spectrum of disorders, from inflammatory diseases to immune deficiencies.

## Materials and methods

### Ethical statement and patient inclusion criteria

All protocols were reviewed and approved by the institutional review boards at Baylor University Medical Center (011-200, 007-221, 012-200), the University of Texas Southwestern Medical Center (092010–167), and the Texas Scottish Rite Hospital (09-11-060). Written informed consent was obtained from adults and the parents or guardians of those younger than 18 yr of age. Children and adolescents with sJIA were enrolled from the Rheumatology Clinic at Texas Scottish Rite Hospital for Children. Clinical laboratory measurements obtained in the clinic at sampling included a CBC, erythrocyte sedimentation rate (ESR), and liver enzymes. Patients with sJIA who were considered “untreated” did not receive any therapy other than nonsteroidal antiinflammatory drugs (NSAIDs). Anakinra-treated patients received the drug within 18 h before blood sampling and were taking no additional medication apart from NSAIDs. The presence of anakinra was confirmed by Luminex analysis of IL1RA (http://tollgene.org). Patients were considered to be inactive based on the physician’s clinical examination and a null MD Global score (a physician’s assessment of overall disease activity with a range 0–10). Inactive untreated patients fulfilled the remission off medication criteria for juvenile idiopathic arthritis including systemic JIA (Wallace et al., 2004). Active patients displayed an MD Global score ≥ 3, arthritis, fever, increased ESR and abnormalities in the CBC. Healthy controls were enrolled at Baylor University Medical Center, did not report any acute or chronic illness, were not receiving immunomodulatory therapies (including over-the-counter allergy medication) apart from NSAIDs, had not received a vaccine at least 1 mo before sampling, and had normal CBC values (measured in-house on a COULTER Ac·T 5diff CP hematology analyzer; Beckman Coulter), and a normal ESR (measured with Winpette Wintrobe ESR Pipets; Arkray). None of the patients whose monocytes were differentiated in vitro had a history of MAS. Patient and healthy control characteristics and inclusion criteria are outlined in Table S5.

### The assay

Ligands were diluted in medium (RPMI 1640 with GlutaMax; Thermo Fisher Scientific) and aliquoted into 2-ml polypropylene 96-well plates (Greiner Bio-One). Blood was drawn in the morning into BD Vacutainers containing ACD anticoagulant (BD). Time between blood draw and culture setup was less than 2 h. Blood was mixed in a 1:1 ratio with medium, added to the diluted ligands in a final volume of 1 ml per well, and incubated for 6 h at 37°C in a 5% CO_2_ atmosphere. After the culture, blood was mixed and a 100-µl aliquot set aside for activation marker phenotyping and intracellular cytokine staining for IL-1β. The plate was then centrifuged at 12,000 *g* for 15 min, and 400 µl plasma was aliquoted and stored at −80°C for Luminex analysis. The pellet was lysed with Tempus solution (Tempus blood RNA tubes; Thermo Fisher Scientific) in a 2:1 ratio and stored at −20°C until the RNA extraction. In case of simultaneous measurement of IL-6, TNF, IL-10, IL-8, and IFN-γ by intracellular cytokine staining, blood was cultured as above in a separate plate containing 1 µg/ml brefeldin A (BFA ready-made solution; Sigma Aldrich) for a total volume of 100 µl. The culture setup was performed in aseptic conditions in BSL-2 cabinet to avoid endotoxin contamination, and all the reagents and labware were designated as pyrogen-free or pyrogen low. The ligands, antibodies, and working concentrations for blood stimulation and phenotyping are listed in Table S1.

### Flow cytometry

#### Whole blood staining

Antibody master mix for surface staining was added to 100 µl blood in Greiner Bio-One plates and incubated for 15 min in the dark at room temperature. Erythrocytes were lysed with 1 ml FACS Lysing Solution (BD Biosciences) for 10 min in the dark at room temperature and then spun down at 600 *g* for 5 min at room temperature. The pellet was washed twice with 1 ml staining buffer (1% fetal calf serum and 0.02% NaN_3_ in PBS, pH 7.4). The cells were fixed with 150 µl 4% formaldehyde overnight at 4°C. Cells were then washed with 1 ml saponin-based Perm/Wash Buffer (BD Biosciences) and incubated with antibodies for intracellular cytokine staining for 30 min at 4°C. The cells were washed again with Perm/Wash buffer, resuspended in staining buffer, and acquired on LSR Fortessa flow cytometer using FACS Diva software (BD Biosciences). The data were analyzed in FlowJo v.9.7.6, and expression values reported as gMFI. gMFI values for each donor were normalized to the unstimulated control.

#### Monocyte staining

Monocytes were stained ex vivo in Falcon 5-ml polystyrene tubes (BD Biosciences) or after culture and supernatant collection in Falcon 96-well flat bottom culture plates (Corning Inc.). First, the cells were labeled with LIVE/DEAD Fixable Yellow Dead Cell dye (50 µl of dye in 1:500 dilution per well; Thermo Fisher Scientific) for 15 min at room temperature in the dark. Cells were then washed twice with staining buffer and processed further as in the whole-blood staining protocol, omitting the erythrocyte lysis step and adjusting volumes for microplate staining.

### Monocyte isolation and stimulation

Monocytes were sorted from frozen PBMCs on a BD Influx cell sorter (BD Biosciences) using the panel described in Table S1. The median purity was 97.1%, and the interquartile range was 4.3. After sorting, cells were lysed ex vivo or resuspended in complete RPMI with 10% FCS at 10^6^ cells/ml and stimulated in 96-well flat-bottom plates for 6 or 24 h ± LPS. Monocyte viability, assessed by FACS, decreased slightly with culture time and stimulation; median viability (interquartile range) ex vivo, at 6 h medium, 6 h LPS, 24 h medium, and 24 h LPS was 98.3% (1.7), 97% (2.2), 93.9% (5.2), 91.7% (4.5), and 90.7% (12.3), respectively. There were no differences in viability between healthy and sJIA monocytes. After incubation, supernatants were collected for Luminex analysis, and cells were either stained for FACS analysis with the monocyte activation panel (Table S1) or lysed with RLT buffer supplemented with 1% β-mercaptoethanol (QIAGEN) for RNA-seq analysis. To confirm that LPS did not adversely affect monocyte viability, lactate dehydrogenase release was measured in the supernatants of LPS-treated monocytes from three healthy controls (Pierce LDH Cytotoxicity Assay Kit; Thermo Fisher Scientific). There was no significant difference in lactate dehydrogenase release between 6-h medium, 6-h LPS, 24 h medium, and 24-h LPS conditions (Kruskal–Wallis ANOVA with Dunn’s post hoc test).

### Monocyte in vitro differentiation

Monocytes were isolated from cryopreserved PBMCs as described above and cultured for 5 d in complete RPMI with 10% FBS at 10^6^ cells/ml in the presence of 100 ng/ml M-CSF, 40 ng/ml of IL-4 (Miltenyi Biotec), and 5 ng/ml TNF (R&D Systems). After culture, cells exhibiting the macrophage or DC phenotype were quantified using flow cytometry staining for CD16 (clone 3G8; BioLegend) or CD1a (clone HI149; BioLegend) markers, respectively. CD163 (clone GHI/61; BioLegend) and viability dye (DAPI) were also included in the staining panel, and the staining buffer was composed of PBS with 0.5% human serum and 2 mM EDTA. For morphological analysis, cells were cytospun, stained with May–Grünwald and Giemsa solutions (Sigma-Aldrich), and imaged using a CFW-1308C color digital camera (Scion Corporation) on a Leica DM 4000 B microscope (Leica Microsystems).

### Multiplex cytokine analysis

For the whole-blood supernatants, samples were diluted 12× using commercial plasma matrix from the kits and then analyzed with the bead-based multiplex 41-human cytokine and chemokine panel (HCYTMAG_60K-PX41) or 29-plex human cytokine and chemokine panel (HCYTMAG-60K-PX29; Luminex Technology; Milliplex; EMD Millipore) according to the manufacturer’s instructions and run on a Bio-Plex 200 reader (Bio-Rad Laboratories). Monocyte supernatants were diluted 10× and assayed for human IL-1β, human IL-6, human IL-10, human TNF-α, and human IP-10 using monoclonal antibody reagent pairs developed and validated in-house. These pairs were conjugated to Luminex beads/biotin and multiplexed together with Millipore MAPmate sets for human IL-1α and human IL-8. This multiplex was assessed using a commercial 68-Plex cytokine standard cocktail produced expressly for the Baylor Institute for Immunology Research by BioLegend with protocols comparable to that for Millipore multiplex processing. The Baylor Institute for Immunology Research Luminex Core facility maintains compliance with the External Quality Assurance Program Oversight Laboratory, a National Institutes of Health and National Institute of Allergy and Infectious Diseases Division of AIDS program for quality assessment review and ratings of laboratories involved in HIV/AIDS research and vaccine trials around the world.

### RNA extraction

RNA was purified from whole-blood cultures using MagMax for Stabilized Blood Tubes RNA Isolation Kit (Thermo Fisher Scientific). Manufacturer’s instructions were followed, but the initial homogenization volumes were proportionally adjusted to the sample input volume. RNA from cell suspensions was purified using the RNAqueous-Micro Total RNA Isolation Kit (Thermo Fisher Scientific). RNA integrity was assessed using RNA 6000 Pico or RNA 6000 Nano assay on Agilent 2100 Bioanalyzer (Agilent Technologies). RNA concentration and purity were assessed using the NanoDrop 8000 (Thermo Fisher Scientific). All procedures were performed according to the manufacturer’s instructions.

### cDNA microarray processing and data analysis

Biotin-labeled cDNA was generated using the Illumina TotalPrep-96 RNA Amplification Kit (Thermo Fisher Scientific) with 250 ng total RNA input for all samples. Labeled cDNA (1.5 µg) was then hybridized onto Illumina Human HT-12 v4 Expression BeadChips (47,231 probes) and scanned on an Illumina Beadstation 500. GenomeStudio software v. 2011.1 with the Gene Expression Module v. 1.9.0 (Illumina) was used to generate signal-intensity values from the scans, subtract background, and rescale the difference in overall intensity to the median intensity across multiple arrays and chips. GeneSpring GX version 7.3.1 (Agilent Technologies) was used to perform further normalization. All signal intensity values less than 10 were set to equal 10. Raw data for all the samples from each donor were normalized to the medium control for that donor, thereby accounting for baseline donor variability and eliminating batch effect (all samples from a donor were run in the same batch). For all datasets, transcripts were filtered out where the detection p-value was greater than 0.01 in all samples, and there was no difference between any groups considered (ANOVA, P < 0.05, Benjamini–Hochberg false discovery rate correction). Data have been deposited in the NCBI Gene Expression Omnibus under accession no. GSE80325.

### Reference module extraction algorithm

Modules were extracted as previously described (Chaussabel et al., 2008). Briefly, coexpression clusters were first identified for each of 15 stimulus groups independently. All pairs of transcripts considered were then assigned a score between 0 and 15 based on the number of stimulus groups in which they coclustered and a weighted cocluster matrix was built. Finally, a graph theory approach organized groups of probes connected by the largest score into cliques, through multiple rounds of selection that go from the most conserved to the most specific clustering pattern across stimulus groups. Each clique defined a module. A transcript could only appear in one module.

### Reference module annotations and predicted regulators

To assess enrichment for interferon-inducible transcripts, we compared the modules with the INTERFEROME database (Rusinova et al., 2013). For each module, we counted the genes induced by type I and type II interferons in the *Homo sapiens* hematopoietic/immune system with twofold up-regulation or 100-fold down-regulation (thereby focusing on transcripts up-regulated by interferons). To identify the putative transcription factors associated with each module, the unique list of gene symbols from each module was selected and subjected to PASTAA analysis (Roider et al., 2009). All transcription factors with P < 0.01 were retained.

### MDTM

The MDTM represents a quantification of the global absolute transcriptional changes in a sample. The MDTM is calculated for each sample as the sum of absolute normalized fold changes ≥2 in the list of transcripts considered.

### WGCNA

The analysis was conducted using the WGCNA R package (Langfelder and Horvath, 2008). For healthy adult blood stimulation, the transcriptional and protein measurements of biological replicates were averaged by stimulus. For sJIA patients, the transcriptional and protein measurements were averaged by the interaction of patient group (healthy, sIU, sIA, and sAU) and stimulus. FACS (gMFI ratio to medium control) and Luminex (fluorescence − background ratio to medium control) data were treated as continuous traits and correlated to the eigengene of each WGCNA module. Transcripts overexpressed at least twofold in response to any stimulus compared with medium control were selected before module extraction. The minimum module size was set to 30, with a merge cut height of 0.1 and a minimum eigengene conectivity to stay of 0.7. WGCNA modules were annotated based on percent overlap with reference modules. When overlaps were low, WGCNA module annotations were conducted by IPA.

### RNA-seq library construction and Illumina sequencing

Total RNA was converted to cDNA using 20 ng RNA as input for the Ovation RNA-Seq System V2, and barcoded Illumina library constructs were produced using Ovation Ultralow Library System V2 (NuGEN Technologies) following the manufacturer’s instructions. Library fragment size and molar concentration were determined using Agilent High Sensitivity DNA Chip on 2100 Bioanalyzer, and quantitative PCR data were obtained by the KAPA BioSystems Library Quantification qPCR Kit for Illumina Platform on a Viia7 Real-Time PCR System (Thermo Fisher Scientific). RNA-seq libraries were sequenced on the Illumina HiSeq 2500 sequencing system using SBS v3 chemistry and two high-output flow cells targeting 70–80 million 2 × 75 paired-end reads per library.

### RNA-seq data processing and analysis

Sequences were aligned with HISAT2 (Kim et al., 2015), duplicates removed with Samtools (Li et al., 2009), and counts generated with HTSeq (Anders et al., 2015) using the annotations from Gencode V20 (Harrow et al., 2012). Genes identified as globins, ribosomal RNAs, and pseudogenes were removed. Differential expression analysis was performed using DESeq2 (Love et al., 2014). For ex vivo analysis, the design matrix was defined by design = ~disease. For 6-h stimulation analysis, the design matrix was defined by design = ~stimulus + disease + stimulus/disease.

### Real-time quantitative PCR

Total RNA was converted to cDNA using SMARTer PCR cDNA synthesis kit and amplified with the Advantage 2 PCR kit (Clontech) and then purified with a QIAquick PCR Purification Kit (QIAGEN). cDNA quantity was determined with a Nanodrop 8000 (Thermo Scientific) and size distribution with the Agilent High Sensitivity DNA kit (Agilent Technologies). Gene expression was measured using the Gene Expression Master Mix (Applied Biosystems) and predesigned TaqMan real-time PCR assays for the following targets: *AHR* (Hs00169233_m1), *HSP90AB1* (Hs00607336_gH), *ACVR2A* (Hs01012007_m1), *IVNS1ABP* (Hs01573482_m1), and *HPRT1* (Hs02800695_m1) on ABI PRISM 7900HT Sequence Detection System (Thermo Scientific). Reactions were set up in duplicate in a 384-well plate with 40 ng cDNA per well in 20 µl volume, and the data were analyzed using RQ Manager 1.2.1 (Thermo Scientific). All experimental procedures were conducted according to the manufacturer’s instructions.

### Activin A ELISA

Supernatants from 6-h and 24-h cultured monocytes were diluted and activin A protein was measured using the Human/Mouse/Rat Activin A Quantikine ELISA Kit (R&D Systems) according to the manufacturer’s instructions.

### Western blot

Monocytes were isolated from cryopreserved healthy adult PBMCs as described above and incubated for 6 and 24 h ±1 ng/ml LPS or 500 IU/ml IFN-α. After culture, supernatants were collected, an aliquot of monocytes was set aside for FACS analysis, and the leftover cells were lysed with Cell Extraction Buffer (Life Technologies) in the presence of Halt Protease & Phosphatase Inhibitor Cocktail (Thermo Fisher Scientific). Protein concentration was determined using the Pierce-BCA Protein Assay kit (Thermo Fisher Scientific). Proteins were denatured in reducing conditions at 95°C for 5 min with 4× Laemmli Sample Buffer supplemented with β-mercaptoethanol (Bio-Rad). Lysates were subjected to SDS-PAGE using 4–15% polyacrylamide stain-free gel (Bio-Rad) and subsequently transferred to 0.2 µm PVDF membrane from the Trans-Blot Turbo Mini PVDF Transfer Pack using the Trans-blot Turbo Transfer System (Bio-Rad). The membrane was blocked for 1 h at room temperature in TBST with 3% BSA, followed by overnight incubation at +4°C with mouse anti–human IL-1β monoclonal antibody (catalog no. MAB201; R&D Systems). The membrane was then washed and incubated with HRP-conjugated anti–mouse IgG secondary antibody for 1 h at room temperature (Cell Signaling Technologies) and developed with chemiluminescence using the ECL Prime detection kit (GE Healthcare). Stain-free and chemiluminescent images were obtained on a ChemiDoc MP imager using Image Lab software (Bio-Rad). All procedures were performed according to manufacturers’ instructions, and protocols were adapted from Bio-Rad’s V3 Western Workflow.

### Statistical methods and data visualization

Using the microarray power calculation tool available at http://bioinformatics.mdanderson.org/MicroarraySampleSize/, we determined that, assuming a 5% false positive rate, a desired fold difference of 2, a desired power of 0.8, and a standard deviation of 0.7, eight replicates per condition would be needed. The median number of replicates per condition in healthy adult blood stimulation is eight. For identification of DETs in microarray experiments, a one-way Welch ANOVA was conducted using a p-value cutoff of 0.05 and Benjamini–Hochberg false discovery rate multiple testing correction. The differences between pairs were analyzed using Tukey’s post hoc test. GeneSpring v.7.3.1. software was used for statistical analysis of microarrays, principal-component analysis, and heatmap plotting. Intracellular and secreted protein levels were compared using the Mann–Whitney *U* test in GraphPad Prism 6.0a (GraphPad Software). Data were plotted with GraphPad Prism and the ggplot2 v2.1.0 graphical package R v. 3.2.1.

### Online data access

The datasets described in this manuscript have been deposited in the NCBI Gene Expression Omnibus under accession no. GSE80325.

### Online supplemental material

Fig. S1 displays in vitro stimulation quality control and module enrichment for genes from the INTERFEROME database. Fig. S2 describes the experimental setup, FACS gating strategy, and protein expression examples. Fig. S3 shows leukocyte activation profiles in whole blood from three healthy adults challenged with 11 stimuli for 6 h. Fig. S4 shows the correlation of transcript, intracellular, and secreted IL-1β in whole blood and isolated monocytes. Fig. S5 displays the FACS gating strategy, activation marker expression, RNA-seq and quantitative RT-PCR of selected genes on isolated healthy and sJIA monocytes, and morphology and CD163 expression on in vitro differentiated monocytes. Tables S1–S5 are included as Excel files. Table S1 lists reagents used for the assay. Table S2 lists the reference modules annotations. Table S3 lists uncataloged IFN-α– and IFN-γ–induced genes. Table S4 lists genes differentially expressed between sJIA and healthy monocytes ex vivo and after 6-h LPS stimulation. Table S5 lists donor characteristics.

## Supplementary Material

Supplemental Materials (PDF)

Tables S1-S5 (zipped Excel files)
