# Supplementary material for: A multidimensional blood stimulation assay reveals immune alterations underlying systemic juvenile idiopathic arthritis
Source: J Exp Med. 2017 Nov 6;214(11):3449–66. doi: 10.1084/jem.20170412 (PMC5679164; doi:10.1084/jem.20170412)
Supplement: Supplemental Materials (PDF) [file JEM_20170412_sm.pdf]

SUPPLEMENTAL MATERIAL

Cepika et al., https://doi.org/10.1084/jem.20170412

The Journal of Experimental Medicine

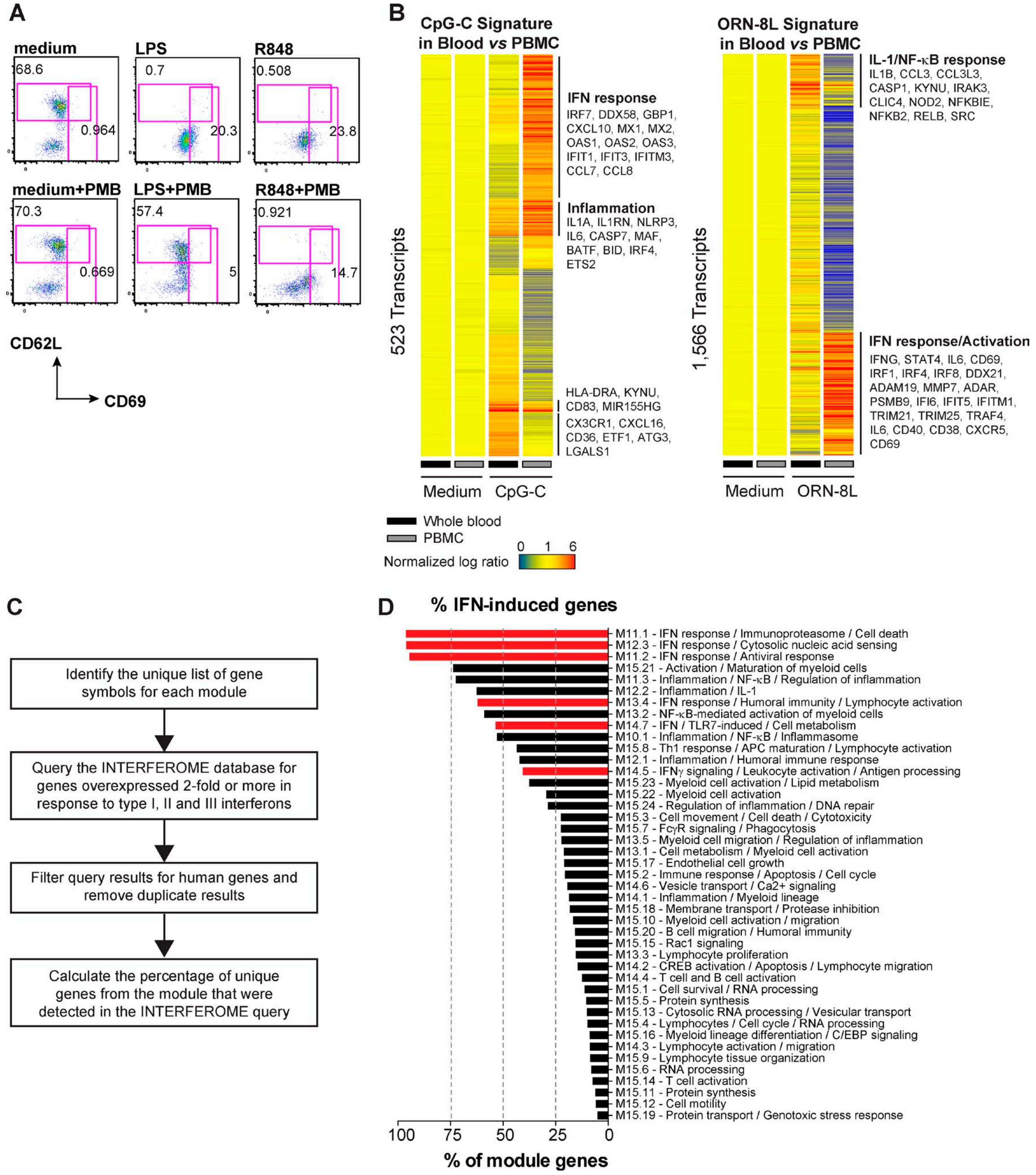

Figure S1. **In vitro stimulation quality control and module enrichment for genes from the INTERFEROME database, related to Fig. 1.** (A) Endotoxin neutralization does not affect R848-induced activation. Blood from three healthy adult donors was stimulated in three independent experiments with LPS and R848 in the presence or absence of polymyxin B (PMB), which binds the lipid A component of LPS (endotoxin) and prevents TLR4 signaling. After 6-h culture, blood was analyzed by flow cytometry. Dot plots display CD62L and CD69 expression on HLA-DR$^+$CD14$^+$ monocytes of one representative donor. Gates (pink quadrants) were set using the medium control. Numbers in gates represent the percentage of total CD14$^+$ monocytes. (B) Oligonucleotides that stimulate TLR8 and TLR9 do not induce an IFN response in whole blood. Whole blood or PBMCs from healthy adult donors were stimulated for 6 h with the TLR9 ligand CpG-C or the TLR8 ligand ORN-8L. Hierarchical clustering of 523 DETs between CpG-stimulated blood and PBMCs (n_{WB} = 8, n_{PBMC} = 3; left). Hierarchical clustering of 1,566 DETs between ORN-8L–stimulated blood and PBMCs (n_{WB} = 6, n_{PBMC} = 4; right). DETs were calculated using Welch *t* test ($P < 0.01$, no multiple testing correction). Representative transcripts from relevant clusters are listed next to the heatmaps. Data are normalized to the medium control for each donor and averaged by stimulus. Whole blood was stimulated as described in Materials and methods. PBMCs were seeded at 0.4 × 10^6 cells per well in RPMI 1640 medium containing 10% FBS. Samples were processed in three or two independent experiments for blood stimulations with CpG or ORN-8L, respectively, and in two independent experiments for PBMCs. (C) Module enrichment analysis workflow. (D) Bar chart representing the percent of INTERFEROME-defined IFN-induced transcripts in each module.

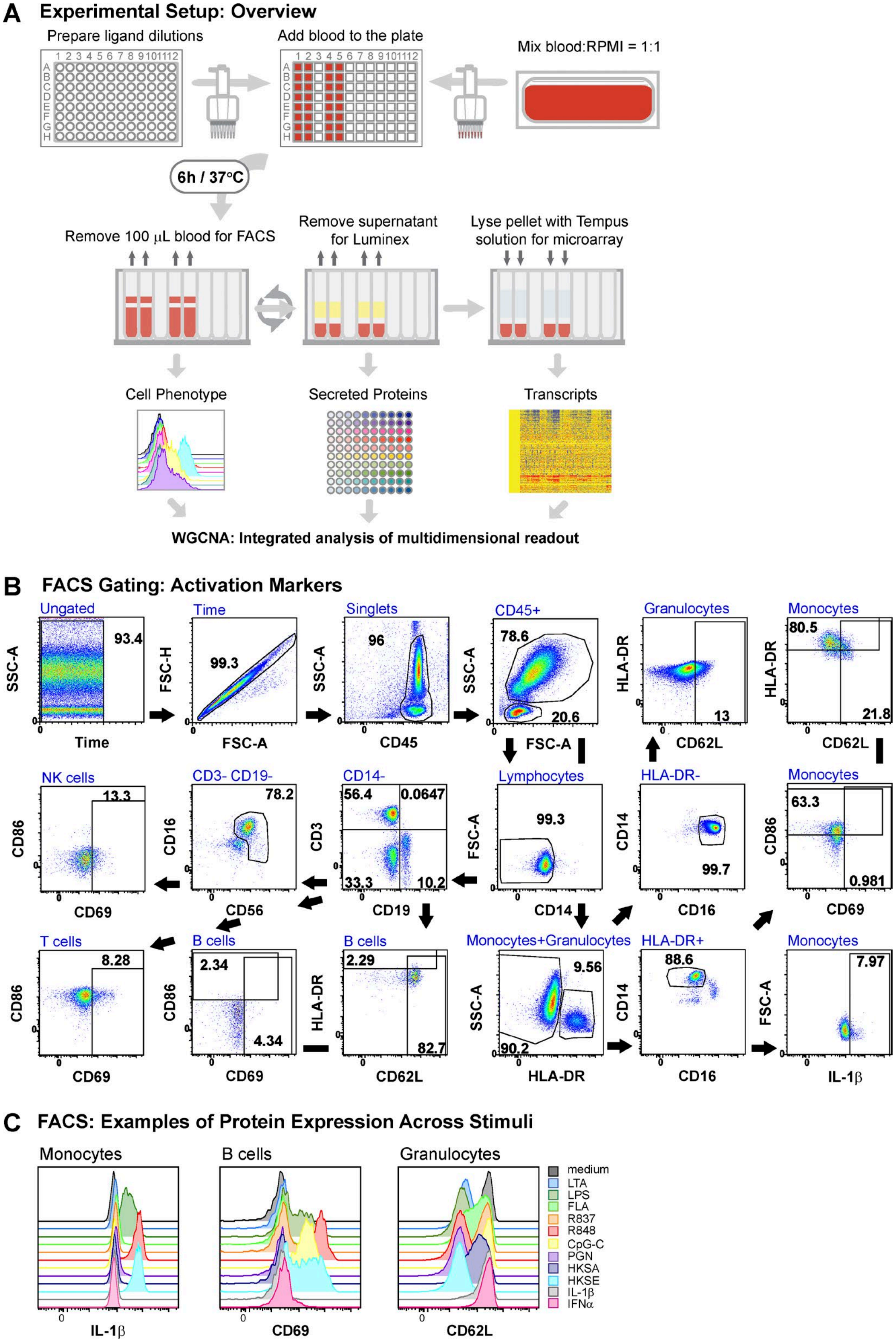

Figure S2. **Experimental setup, FACS gating strategy, and protein expression examples, related to Fig. 2.** (A) Schematic workflow of the expanded in vitro stimulation assay. (B) After 6-h stimulation, T cells, B cells, NK cells, monocytes, and neutrophils were examined for expression of activation markers. Plots depict peptidoglycan (PGN) stimulation of a representative healthy adult donor. Gates were set according to the medium control. Labels on top of the plots represent the parent gate. (C) Expression of IL-1β in monocytes, CD69 on B cells, and CD62L on granulocytes from one donor across all stimuli.

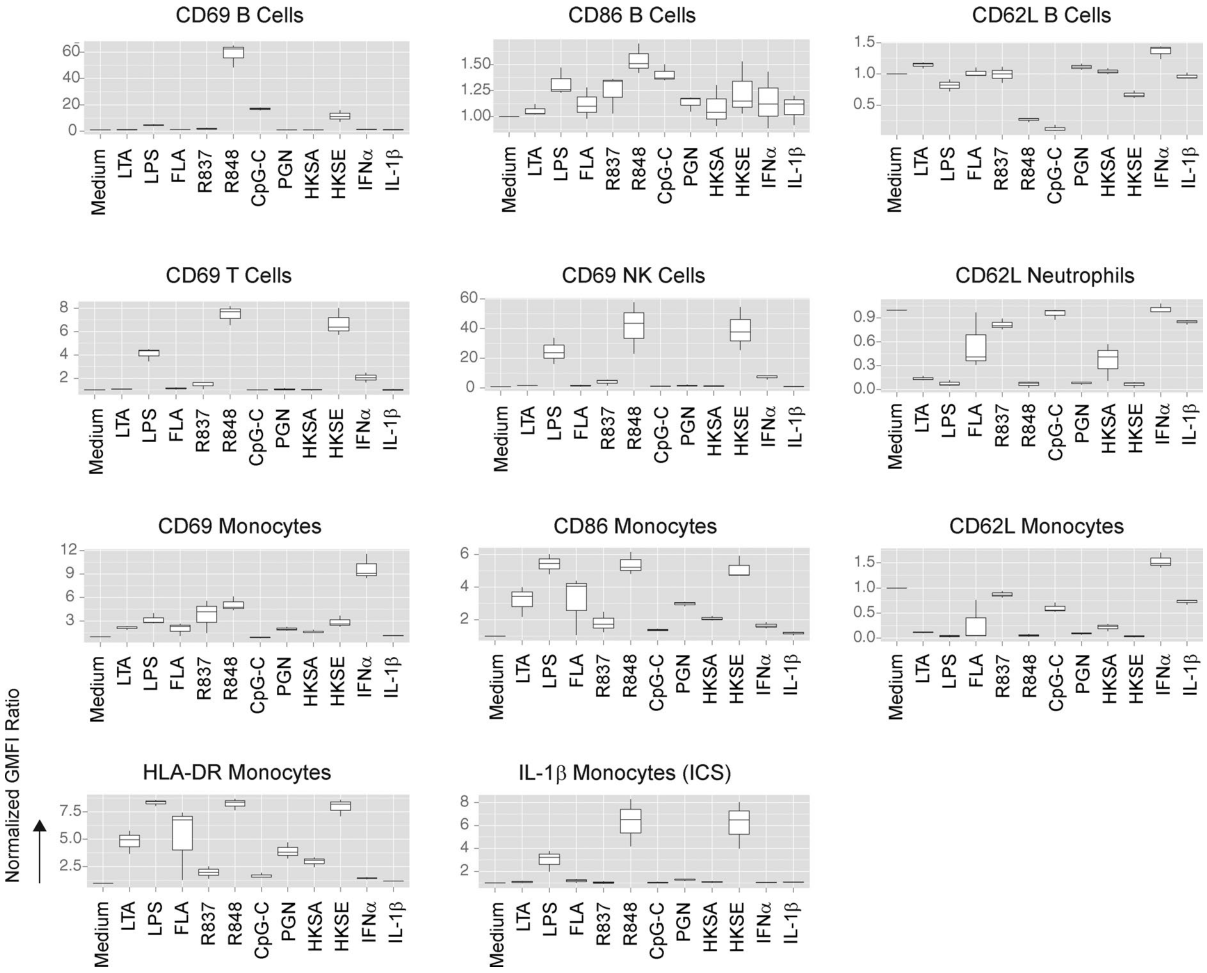

Figure S3. **Leukocyte activation profiles in whole blood from three healthy adults challenged with 11 stimuli for 6 h, related to Fig. 2.** Bar charts represent the ratio of gMFI for each stimulus over the medium control sample. Horizontal lines represent the median. Boxes represent the interquartile range, and whiskers the nonoutlier range. HKSA, heat-killed *Staphylococcus aureus*; HKSE, heat-killed *Salmonella enterica*.

A

Dataset	Comparison	Spearman *r*	*P* value	n (xy pairs)
Healthy Adult Controls	ICS_sup	0.9	<0.0001	35
	ICS_trx	0.79	<0.0001	35
	trx_sup	0.78	<0.0001	35
Healthy Pediatric Controls	ICS_sup	0.87	<0.0001	53
	ICS_trx	0.48	0.0002	53
	trx_sup	0.34	0.0122	53
sJIA Inactive Untreated	ICS_sup	0.89	<0.0001	36
	ICS_trx	0.87	<0.0001	36
	trx_sup	0.85	<0.0001	45
sJIA Inactive Anakinra	ICS_sup	0.88	<0.0001	42
	ICS_trx	0.8	<0.0001	42
	trx_sup	0.73	<0.0001	45
sJIA Active Untreated	ICS_sup	0.87	<0.0001	18
	ICS_trx	0.77	0.0002	18
	trx_sup	0.89	<0.0001	18

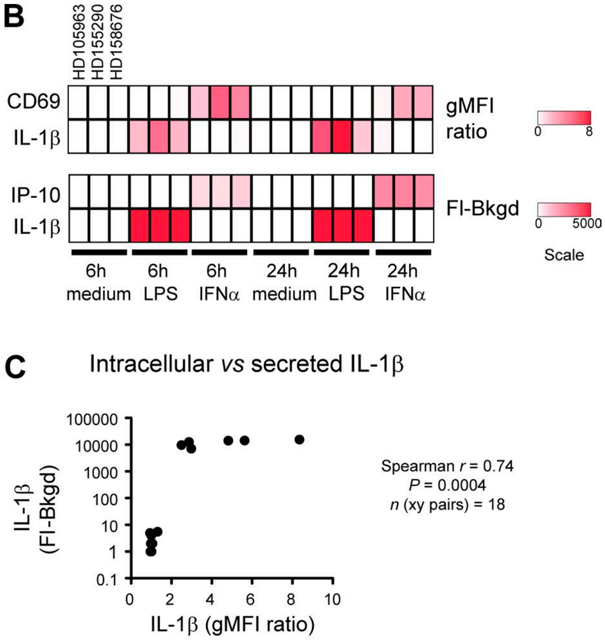

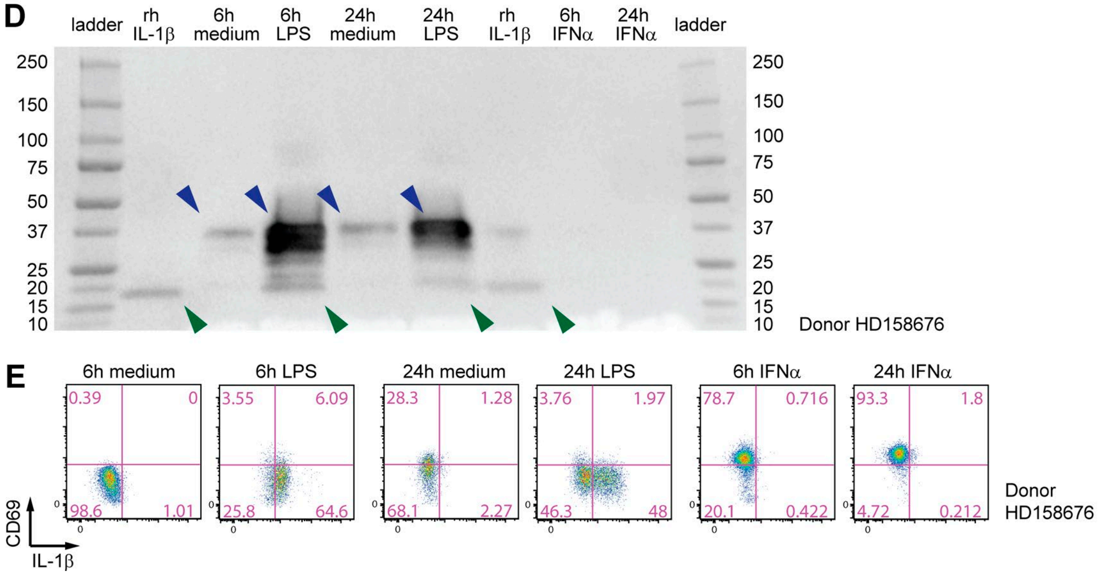

Figure S4. **Levels of transcript, intracellular and secreted IL-1b correlate in whole blood and isolated monocytes, related to Fig. 3.** (A) Spearman rank-order correlations between intracellular IL-1β (intracellular cytokine staining [ICS], measured by flow cytometry), secreted IL-1β ("sup"; Luminex) and *IL1B* transcript ("trx"; microarray) in stimulated whole blood. All comparisons are statistically significant ($P < 0.05$). (B) Secreted and intracellular IL-1β were measured in culture of sorted $CD14^{+}CD16^{-}$ monocytes from three healthy adults stimulated for 6 h and 24 h with 1 ng/ml LPS or 500 IU/ml IFN-α. IFN-α served as a control stimulus that activates monocytes without triggering the inflammasome. Monocytes from each donor were cultured independently. (C) Intracellular and secreted IL-1β in sorted monocytes positively correlate. (D) Western blot analysis of mature and pro–IL-1β expression in sorted monocytes from a representative donor from the dataset depicted in B and C. The pro–IL-1β band is expected to migrate at 31 kD (blue arrowheads) and the mature IL-1β band at 17 kD (green arrowheads). Recombinant human 17-kD IL-1β (rh IL-1β) added at 5 ng was used as a positive control for staining. (E) Flow cytometry analysis of intracellular IL-1β from the same donor. FI-Bkgd, fluorescence − background.

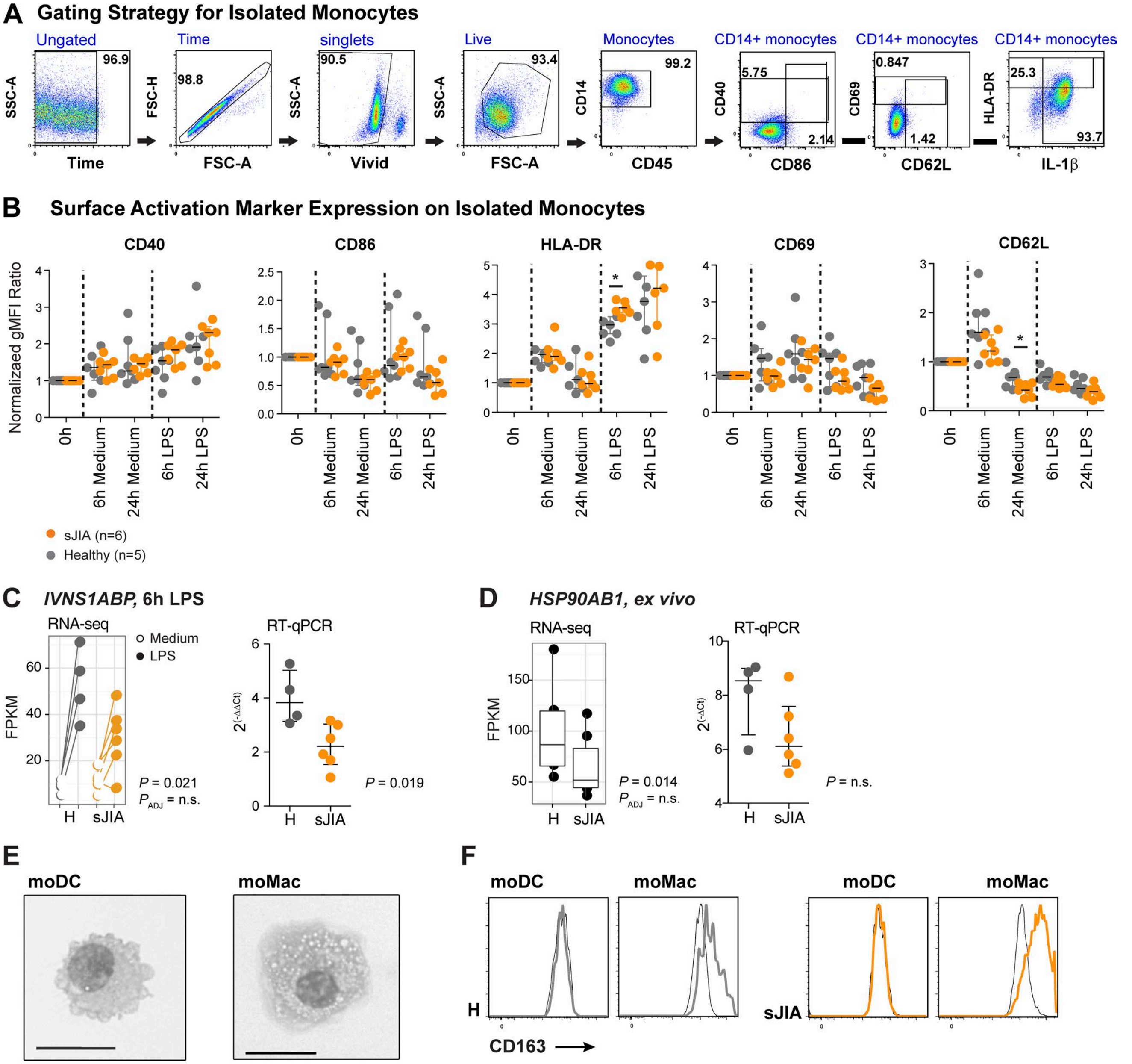

Figure S5. **FACS gating strategy and activation marker expression on sorted monocytes, RNA-seq and quantitative RT-PCR of selected genes, and morphology and CD163 expression on in vitro differentiated monocytes, related to Fig. 6.** (A) Gating strategy for sorted $CD14^{+}CD16^{-}$ monocytes. Representative plots for monocytes from one donor stimulated with 1 ng/ml LPS for 24 h. Gates were set according to the unstimulated control. Numbers on the plots represent percent of the parent gate, indicated on top of the plots. (B) Scatter dot plots representing the normalized gMFI ratios for monocyte activation markers CD40, CD86, HLA-DR, CD69, and CD62L. Data were normalized to the baseline (0 h) sample for each donor. (C) Expression of *IVNS1ABP* gene in healthy control and sJIA patient monocytes after 6-h culture in medium or with 1 ng/ml LPS. RNA-seq data expressed as FPKM (left). Quantitative PCR data expressed as $2^{-\Delta\Delta Ct}$ (right). (D) Expression of *HSP90AB1* gene in healthy controls and sJIA patients monocytes ex vivo. RNA-seq data expressed as FPKM (left). Quantitative RT-PCR data expressed as $2^{-\Delta Ct}$ (right). (E) Primary human monocytes were differentiated in vitro for 5 d, sorted based on $CD1a^{+}$ and $CD16^{+}$ markers into monocyte-derived dendritic cells (moDCs) and monocyte-derived macrophages (moMac), respectively, and imaged after cytospin and May–Grünwald–Giemsa staining. Bars, 10 µm. Representative of five independent experiments on healthy adult monocytes. (F) Representative histograms of CD163 expression on differentiated monocytes from one healthy control and one sJIA patient after 5 d of culture, gated through dendritic cell (moDC, $CD1a^{+}$) or macrophage gate (moMac, $CD16^{+}$). H, healthy control; sJIA, inactive untreated sJIA patient. Horizontal lines represent the median and whiskers interquartile range. P-values for RNA-seq data were calculated using DESeq2 package. P-values for flow cytometry and quantitative RT-PCR data were calculated using Mann–Whitney *U* test. *, $P < 0.05$.

Tables S1–S5 are included as Excel files. Table S1 lists reagents used for the assay. Table S2 lists the reference modules annotations. Table S3 lists uncataloged IFN-α– and IFN-γ–induced genes. Table S4 lists genes differentially expressed between sJIA and healthy monocytes ex vivo and after 6-h LPS stimulation. Table S5 lists donor characteristics.
